# Anti-miR-203 suppresses ER-positive breast cancer growth and stemness by targeting SOCS3

**DOI:** 10.18632/oncotarget.11193

**Published:** 2016-08-10

**Authors:** Naoshad Muhammad, Sourav Bhattacharya, Robert Steele, Ratna B. Ray

**Affiliations:** ^1^ Department of Pathology, Saint Louis University, St. Louis, Missouri, USA; ^2^ Cancer Center, Saint Louis University, St. Louis, Missouri, USA

**Keywords:** breast cancer, miR-203, SOCS3, stemness

## Abstract

Breast cancer is a major public health problem worldwide in women and existing treatments are not adequately effective for this deadly disease. microRNAs (miRNAs) regulate the expression of many target genes and play pivotal roles in the development, as well as in the suppression of many cancers including breast cancer. We previously observed that miR-203 was highly upregulated in breast cancer tissues and in ER-positive breast cancer cell lines. In our present study, we observed that anti-miR-203 suppresses breast cancer cell proliferation *in vitro*. Orthotopic implantation of miR-203 depleted MCF-7 cells into nude mice displays smaller tumor growth as compared to control MCF-7 cells. Furthermore, miR-203 expression is significantly higher in ER-positive breast cancer patients as compared to ER-negative patients. We identified suppressor of cytokine signaling 3 (SOCS3) as a direct target of miR-203. Here we observed that miR-203 expression is inversely correlated with SOCS3 expression in ER-positive breast cancer samples. Additionally, we found that anti-miR-203 suppressed the expression of pStat3, pERK and c-Myc in MCF-7 and ZR-75-1 cells. We also demonstrated that anti-miR-203 decreased mammospheres formation and expression of stem cell markers in MCF-7 and ZR-75-1 cells. Taken together, our data suggest that anti-miR-203 has potential as a novel therapeutic strategy in ER-positive breast cancer treatment.

## INTRODUCTION

Breast cancer is one of the most widespread cancer related malignancy among women throughout the world and the most common cause of cancer-related deaths. In the United States, 292,130 women were diagnosed with breast cancer and among them 40,290 died from this deadly disease in 2015 (American Cancer Society: Breast cancer facts and figures 2015-2016). Recent studies suggest that tumors consist of heterogeneous mass of cells, which retain diverse biological functions. The potential for development of tumor and its progression resides solely in a small population of cancer cells, termed as cancer stem cells (CSCs). This population of cells can self-renew and re-form the ordered organization of tumors [1]. CSCs have been identified in all types of solid tumors including breast cancer [2]. Accumulating evidence demonstrated that CSCs added to several characteristics of tumor pathogenesis such as initiation of tumor, metastasis and recurrence [3].

MicroRNAs (miRNAs) are a highly conserved class of non coding small RNAs. They have a size ranging from 20 to 25 nucleotides and regulate the expression of particular key genes which are implicated in various types of biological processes such as self-renewal, survival, and tumor progression [4–6]. In various studies, several miRNAs, such as miR-451, miR-128, and miR-34, apparently regulate cancer stemness and drug resistance in breast cancer [7]. miRNAs play a crucial role in self-renewal of CSCs. miR-100 suppressed the maintenance and expansion of CSCs in breast cancer, and ectopic expression of this miRNA enhanced CSC differentiation [8, 9]. We have shown previously that miR-203 is highly expressed in breast cancer tissues and ER-positive breast cancer cell lines [10]. Further, clinical data suggested that miR-203 is highly expressed in ER-positive breast cancer tissues [11]. SOCS3 was identified as a direct target of miR-203 [10]. Cytokine dependent activation of Stat3 is very crucial for a wide range of physiological processes via IL6/NFkB mediated signaling [12]. However, continuous activation of this axis exhibits cell survival, proliferation and metastases of breast cancer cells [13]. One of the most important genes induced in response to Stat3 mediated signaling is SOCS3 [14]. It is the member of a family of SOCS proteins and negatively modulates cytokine signal transduction via binding with phosphotyrosine residues of cytokine receptors, and suppresses receptor-associated JAK activity [15]. SOCS3 is targeted by several miRNAs in different pathological conditions [10, 16–18].

In the present study, we investigated the role of miR-203 in breast cancer cell growth regulation. We observed that anti-miR-203 in breast cancer cells alters tumor growth. Subsequently, we studied whether knockdown of miR-203 plays a role in the stemness of breast cancer cells. Our results demonstrate that the miR-203-SOCS3 axis plays an important role in breast cancer growth by inhibiting spheroid formation and CSC marker expression. Our findings may offer to develop a novel miRNA based therapy (using miR-203 inhibition) for management of ER-positive breast cancer.

## RESULTS

### Anti-miR-203 suppresses cell proliferation of breast cancer cells

**Figure 1:**
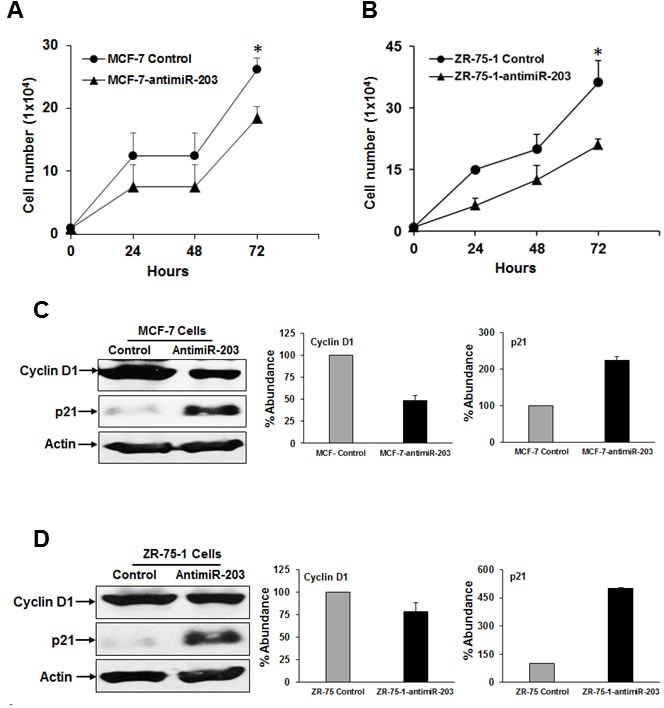
Inhibition of miR-203 expression alters cell proliferation and key cell cycle regulatory proteins **A.** MCF-7-control and MCF-7-antimiR-203 cells, **B.** ZR-75-1-control and ZR-75-1-antimiR-203 cells were plated in 35 mm plates in triplicates, and live cells were counted at indicated time points by using Trypan blue exclusion method. Small bar indicates standard error (*, *p* < 0.05). **C.** MCF-7-control and MCF-7-antimiR-203 cells, **D.** ZR-75-1-control and ZR-75-1-antimiR-203 cell lysates were subjected to Western blot analysis using indicated antibodies. These blots were reprobed with an antibody to actin for comparison of protein loading in each lane. Densitometric analysis of cyclin D1 and p21 were done by using Image J software and shown on the right. Data are represented as mean ± SD.

One of the key features of cancer cells is uncontrolled proliferation. In order to investigate the role of miR-203 in breast cancer, cell growth was examined by Trypan blue exclusion method and cells were counted. We observed that inhibition of miR-203 caused a significant decrease in the rate of cell proliferation in MCF-7-antimiR-203 and ZR-75-1-antimiR-203 cells as compared to control cells (Figure 1, panels A & B). To investigate whether miR-203 mediated cell proliferation involves the expression of key cell cycle regulatory proteins, we performed Western blot analysis of cyclin D1 and p21. Our results showed that anti-miR-203 decreased cyclin D1 and increased p21 expression levels in MCF-7-antimiR-203 and ZR-75-1-antimiR-203 cells as compared to control cells (Figure 1, panels C & D). Collectively, these findings suggested that anti-miR-203 decreased the proliferation of breast cancer cells by inhibiting cyclin D1 and enhancing p21 expression.

### Anti-miR-203 increases the expression of SOCS3 and decreases pStat3 expression in breast cancer cells

**Figure 2:**
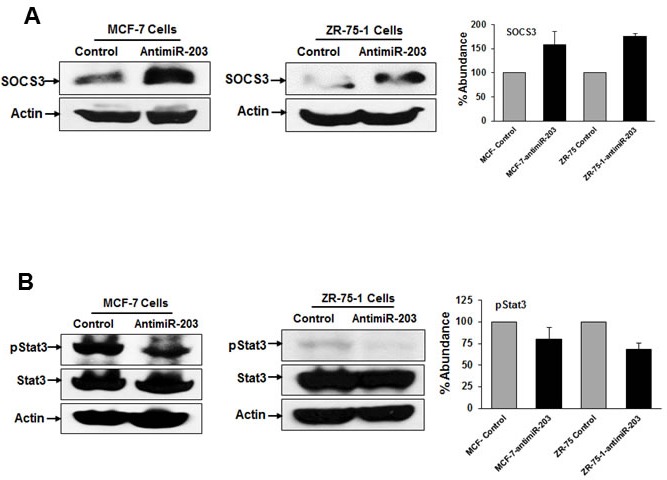
Inhibition of miR-203 increases the expression of SOCS3 and enhances phospho-STAT3 expression in breast cancer cells MCF-7 control, MCF-7-antimiR-203, ZR-75-1 control and ZR-75-1-antimiR-203 cells lysates were subjected to Western blot analysis using SOCS3 (**A**), pStat3 and total Stat3 (**B**) antibodies. The blots were reprobed with an antibody to actin for comparison of protein loading in each lane. Densitometric analyses of all these proteins were done by using Image J software and shown on the right. Data are represented as mean ± SD.

Our previous study have identified that SOCS3 is a direct target of miR-203 [10]. SOCS3 is known to be down-regulated in various types of cancers including breast cancer [19, 20]. We observed that SOCS3 expression was increased in the MCF-7-antimiR-203 and ZR-75-1-antimiR-203 cells as compared to control cells (Figure 2, panel A). SOCS3 is regulated by Stat3 via the IL6/Stat3/NFkB mediated signaling pathway and SOCS3 negatively regulates the expression of Stat3 [21]. Therefore, we examined the expression of pStat3 in breast cancer cells. We observed that the level of pStat3 was reduced in both MCF-7-antimiR-203 and ZR-75-1-antimiR-203 cells as compared to control cells (Figure 2, panel B). However, total Stat3 expression remained unchanged. These data suggested that miR-203 inhibits SOCS3 expression and concurrently increased the expression of pStat3 in these breast cancer cells.

### Anti-miR-203 decreases the expression of pERK and c-Myc in breast cancer cells

**Figure 3:**
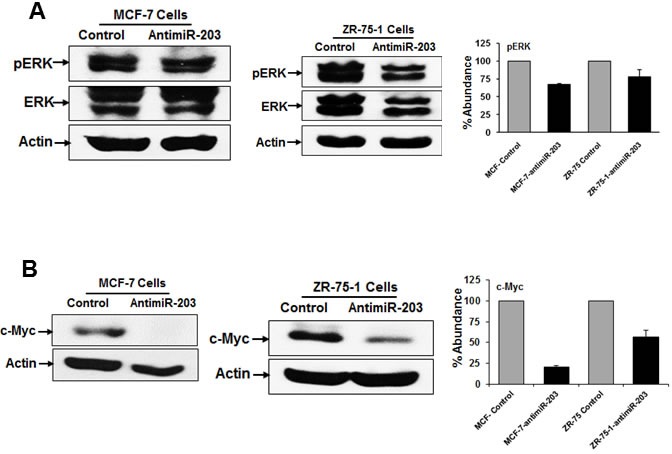
ERK-c-myc signaling pathways are inhibited by anti-miR-203 in breast cancer cells MCF-7 control, MCF-7-antimiR-203, ZR-75-1 control and ZR-75-1-antimiR-203 cells lysates were analyzed for pERK, ERK (**A**) and c-Myc (**B**) expression by Western blot analysis. The blots were reprobed with an antibody to actin for comparison of protein loading in each lane. Densitometry analyses of all these proteins were done by using Image J software and shown on the right. Data are represented as mean ± SD.

Activated ERK is associated with differentiation and proliferation of cells in various types of cancers including breast cancer [22]. We examined the expression of pERK by Western blot analysis. We observed that the pERK was decreased in both MCF-7-antimiR-203 and ZR-75-1-antimiR-203 cells as compared to control cells (Figure 3, panel A). Interestingly, total ERK expression remained unchanged in MCF-7 cells whereas decreased in ZR-75-1 cells and reason is unknown at present. c-Myc is an essential transcription factor that has been extensively studied due to its vital functions in the regulation of cancer cell growth [23]. It also plays a critical role in tumor initiation, progression, and survival of cancer [24]. Therefore, we examined the expression of c-Myc in breast cancer cells by Western blot analysis. We observed that c-Myc expression was decreased in both MCF-7-antimiR-203 and ZR-75-1-antimiR-203 cells as compared to control cells (Figure 3, panel B).

### Inhibition of miR-203 decreases tumor growth in the nude mice

**Figure 4:**
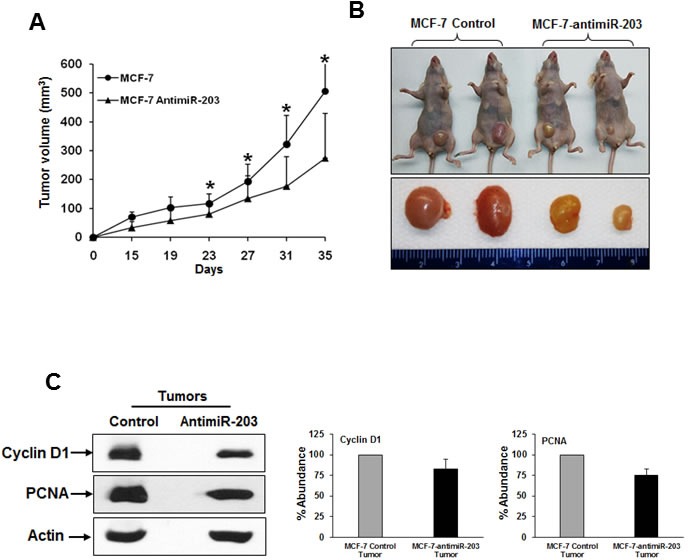
Inhibition of miR-203 in MCF-7 cells nude mice reduces tumor growth in nude mice **A.** MCF-7-control and MCF-7-antimiR-203 cells were implanted into the mammary fat pad of athymic nude mice. Volume of tumor growth was monitored as indicated times and presented as a mean. Small bar indicates standard error (*, *p* < 0.05). **B.** Representative mice image and their respective tumors dissected from control (MCF-7-control) and experimental (MCF-7-antimiR-203) group of mice are shown. **C.** Western blot of cyclin D1 and PCNA in xenograft tumor tissues isolated from both control and experimental mice. The blot was reprobed with an antibody to actin for comparison of protein load. Densitometry analyses of these proteins were done by using Image J software and shown on the right. Data are represented as mean ± SD.

Our *in vitro* data revealed that miR-203 expression is associated with proliferation of breast cancer cells. We next investigated whether inhibition of miR-203 could also suppress tumor progression *in vivo*. Control MCF-7 and MCF-7-antimiR-203 cells (5×10^6^) were injected orthotopically into nude mice. Tumor size was significantly smaller from MCF-7-antimiR-203 cell implantation as compared to control MCF-7 cells (Figure 4, panels A & B). Tumors were extracted from sacrificed the mice, and tumor lysates were used for biochemical evaluation. Our *in vitro* data suggested that cyclin D1 expression was inhibited in miR-203 knockdown breast cancer cells as compared with control cells (Figure 1). We further examined the expression level of cyclin D1 and PCNA by Western blot analysis. Our results demonstrated that the expression of cyclin D1 and PCNA was significantly lower in tumors from miR-203 knockdown MCF-7 cells as compared to control cells (Figure 4, panel C). Thus, these results indicated that miR-203 inhibition plays a role, in part, for reduction of MCF-7 tumor growth.

### miR-203 and SOCS3 are inversely expressed in ER-positive breast cancer tissues

**Figure 5:**
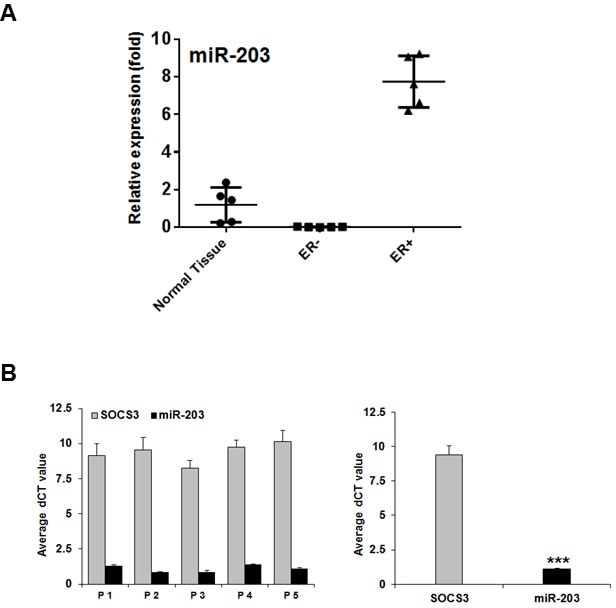
miR-203 suppresses SOCS3 in ER-positive breast cancer tissues **A.** Total RNA was extracted from the ER-positive and the ER-negative breast cancer tissues. Normal breast tissues were included as control. miR-203 expression was analyzed by real-time PCR, using U47 RNA as an internal control. A comparison of miR-203 levels in normal breast tissues, ER-positive and ER-negative breast cancer tissues (*n* = 5) are shown using scattered plot. (***, *p* < 0.0001) **B.** Expression of miR-203 and SOCS3 was examined from ER-positive breast cancer tissues (*n* = 5). For SOCS3 expression, samples were normalized with 18S RNA. We presented SOCS3 and miR-203 expression (dCT value) from individual patient tissue sample and mean expression. A strong inverse expression between miR-203 and SOCS3 levels RNA levels was noted. (***, *n* < 0.0001).

We further examined the expression of miR-203 and SOCS3 in limited breast cancer tissues. RNA from ER-positive breast cancer tissues displayed altered miR-203 expression levels compared to those of ER-negative breast cancer tissues (Figure 5, panel A). We also compared with normal breast tissues. We observed that miR-203 expression is higher in ER-positive breast cancer as compared to normal tissue, but lower in ER-negative breast cancer tissues. The miR-203 expression level was inversely related to the expression level of SOCS3 in ER-positive breast cancer tissues (Figure 5, panel B). Although we have used limited number of samples, the difference is statistically highly significant. Further, our results are in agreement with the earlier miR-203 expression data in primary human breast cancer tissues [10, 11]. Together, our results suggested that miR-203 inhibits SOCS3 expression in the ER-positive breast cancer tissues.

### Inhibition of miR-203 expression reduces breast cancer stemness

**Figure 6:**
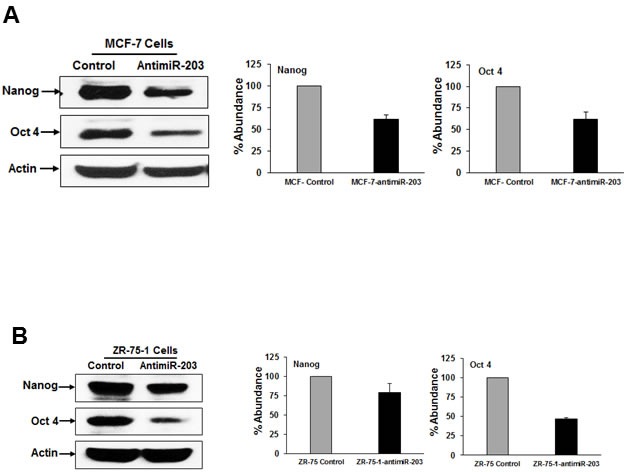
Inhibition of miR-203 reduces the expression of stemness makers **A.** MCF-7 and MCF-7-antimiR-203 **B.** ZR-75-1 and ZR-75-1-antimiR-203 cell lysates were subjected to Western blot analyzing using Nanog and Oct 4 antibodies. These blots were reprobed with an antibody to actin for comparison of protein loading in each lane. Densitometric analyses of these proteins were done by using Image J software and shown on the right. Data are represented as mean ± SD.

**Figure 7:**
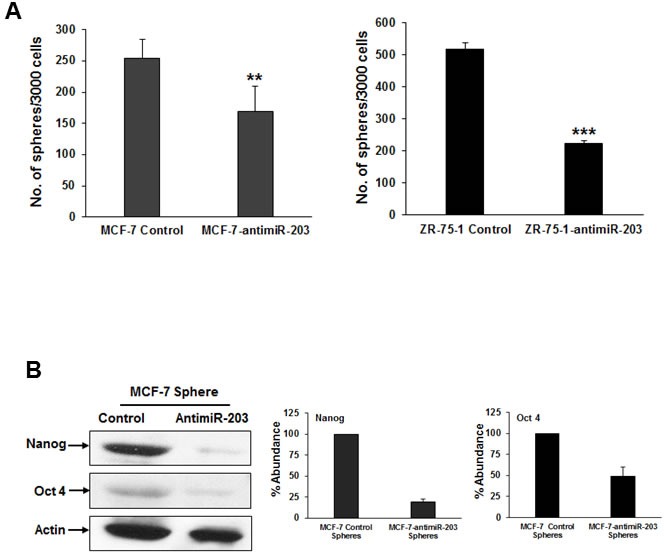
Inhibition of miR-203 reduces mammosphere formation and the expression of Nanog and Oct4 **A.** Equal number of MCF-7-control, MCF-7-antimiR-203, ZR-75-1-control and ZR-75-1-antimiR-203 cells were seeded in ultra-low attachment plates and incubated for 10 days. The number of spheres (>70 mm) were counted. The results are presented as means of three different experiments with standard deviations (**p < 0.01, ***p < 0.001). **B.** Mammospheres lysates from MCF-7-control and MCF-7-antimiR-203 cells were analyzed for expression of Nanog and Oct 4 using specific antibodies. These blots were reprobed with an antibody to actin for comparison of protein loading in each lane. Densitometric analyses of these proteins were done by using Image J software and shown on the right. Data are represented as mean ± SD.

Breast cancer stem cells augment the tumor-initiating ability and are highly drug resistant as compared to cancer cells [3]. Cancer stem cells are regulated by several miRNAs [7]. To investigate the role of miR-203 in breast cancer stemness, we examined the expression of Nanog and Oct4 (stem cell markers) in control or MCF-7-antimiR-203 and ZR-75-1-antimiR-203 cells by Western blot analysis. We observed that the protein level of Nanog and Oct4 were significantly downregulated in both MCF-7-antimiR-203 and ZR-75-1-antimiR-203 cells as compared to control cells (Figure 6, panels A & B). We next examined the effect of miR-203 inhibition in the self-renewal capacity of breast cancer stem cells using a well-established mammosphere formation assay. An equal number of control and experimental cells were seeded in ultra-low attachment plates and incubated for 10 days. The number of mammospheres was significantly lower in anti-miR-203 expressing cells as compared to control cells (Figure 7, panel A). Interestingly, mammospheres from control cells were larger in size and grew more rapidly as compared to those generated from anti-miR-203 expressing cells. We also examined the expression level of Nanog and Oct4 in mammospheres from control and experimental cells. Western blot data indicated that the anti-miR-203 downregulates the expression of Nanog and Oct4 in the mammospheres of MCF-7 cells (Figure 7, panel B). These results suggested that miR-203 plays a role in self-renewal capacity of breast cancer stem cells.

## DISCUSSION

In this study, we observed that inhibition of miR-203 in ER-positive breast cancer cells suppresses cell proliferation by inhibiting cyclin D1 and pStat3, and inhibits tumor growth in a breast cancer preclinical model. Interestingly, we did not observe significant miR-203 expression in ER-negative human breast cancer cells tested. We also observed that SOCS3 expression was lower in ER-positive breast cancer tissues. Dysregulation of miRNA expression is associated with various human diseases including cancer. Additionally, many miRNAs contribute in tumor initiation and progression by targeting various mRNA expressions and may act as oncogenes or tumor suppressors [25]. Therefore, ectopic expression or inhibition of particular miRNAs associated with cancer may be an attractive therapeutic target.

Cancer stem cells play a pivotal role in tumor initiation, maintenance, metastasis and resistance to therapy. Breast cancer stem cells are not the exception. Studies have shown that breast-CSCs display enhanced ability to form tumors [2, 26]. CSCs are implicated in tumor initiation and progression and contribute to radiotherapy and chemotherapy resistance in the patients having cancer [3, 27]. Therefore, understanding the molecular mechanisms that drive CSCs is crucial for the improvement of novel therapeutic strategies against breast cancer. Several miRNAs (such as miR-21, miR-155, miR-10b, miR-221/222) play a critical role in breast cancer including cell proliferation, invasion, angiogenesis, and breast cancer metastasis [28–30]. However, the role of miR-203 in breast cancer stemness was never been studied. In this study, we observed that inhibition of miR-203 reduces mammosphere formation and cancer stem cell marker expression.

miR-203 has been proposed as a tumor suppressor in leukemia stem cells because it targets various oncogenes such as Src, survivin and Bmi-1 [31]. On the other hand, recent report showed that miR-203 expression increased the proliferation, migration and invasion of pancreatic cancer cells [32]. Therefore, miR-203 may exert both tumor suppressor and tumor promoting functions depending on the cellular milieu.

**Figure 8:**
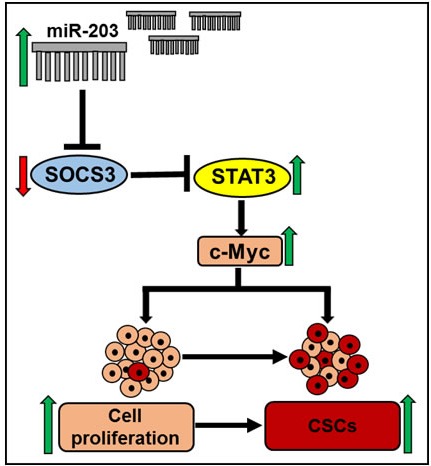
Schematic diagram of proposed mechanism miR-203 expression may regulate breast cancer cells proliferation and stemness by inhibiting the expression of SOCS3 and enhancing expression of Stat3 and c-Myc.

In this study, we demonstrated that miR-203 acts like an oncomiR and its expression potently targets tumor suppressor protein, SOCS3 and concurrently activates pStat3, pERK and c-Myc which enhance the proliferation and stemness of ER-positive breast cancer cells. However the relationship of ER positive and miR-203 upregulation remains to be elucidated. There is estrogen responsive element in miR-203 promoter sequences [33]. Therefore, it is possible that ER transcriptionally enhances miR-203 in ER-positive breast cancer cells. Indeed, further study is needed to understand the underlying mechanism. Our findings are also an agreement with other study that miR-203 expression is upregulated in primary breast tumors [11]. Based on our results, we proposed a novel molecular mechanism of miR-203 in regulation of breast cancer cell growth and stemness (Figure 8). Further study needs to be focused on the miR-203-SOCS3 axis for development of additional therapeutic approaches against breast cancer. To our knowledge, this is the first study showing the role of miR-203 in ER-positive breast cancer stemness.

## MATERIALS AND METHODS

### Cell lines

Breast cancer cell lines (MCF-7 and ZR-75-1) were purchased from the American Type Culture Collection (ATCC) and maintained in RPMI-1640 (Sigma) medium supplemented with 10% fetal bovine serum (FBS), and 1% penicillin/streptomycin in a humidified CO_2_incubator. For stable cell line preparation, MCF-7 and ZR-75-1 cells were transduced with replication-deficient lentivirus expressing anti-miR-203 and control miR. Cells were selected with puromycin, pooled and established a stable cell lineas described previously [10].

### Cell proliferation assay

Cells (MCF-7-control, MCF-7-antimiR-203, ZR-75-1-control and ZR-75-1-antimiR-203) were seeded at 1×10^4^ in 35 mm plates. After 24, 48 and 72 h, the cells in each plate were trypsinized. Cells were counted in triplicates by Trypan blue exclusion method using a haemocytometer.

### Sphere (mammosphere) formation assay

For mammosphere culture, MCF-7-control, MCF-7-antimiR-203, ZR-75-1-control and ZR-75-1-antimiR-203 cells (3000 cells/well) were seeded in single cells suspension on ultralow attachment plates (Corning). Cells were grown in a serum-free growth medium supplemented with EGF and FGF. After 10 days of incubation, numbers of spheres which were >75 μm diameter were counted using vertical microscope (Leica).

### Western blot analysis and antibodies

Cell lysates were prepared from adherent and mammosphere cells derived from MCF-7-control, MCF-7-antimiR-203, ZR-75-control and ZR-75-1-antimiR-203 cell lines, analyzed by SDS-PAGE and transferred onto 0.45 μM nitrocellulose membrane (Bio-Rad). Membranes were blocked using 5% low fat dry milk in TBST and probed with the respective primary antibodies. Proteins were detected using ECL Western Blotting Substrate (Thermo Scientific) and autoradiography. The protein loading was normalized using antibody to β-actin. The following antibodies were used in this study: Nanog, Oct4, c-Myc, pERK, ERK, pStat3 and Stat3 (Cell Signaling Technologies), SOCS3 and β-actin (Santa Cruz Biotechnology).

### *In vivo* studies

Animal experiments were performed according to the NIH guidelines, following a protocol approved by the Institutional Animal Care and Use Committee (IACUC) of Saint Louis University. Nude mice (6 week old females) were purchased from Charles River, and housed in a specific pathogen free animal facility at the Saint Louis University. Each mice was implanted with a 0.72 mg of 17β-estradiol pellet (90 days release, Innovative Research of America) to supplement the estrogens required for MCF-7 proliferation as described previously [34]. One day after implantation of 17β-estradiol pellet, 5×10^6^viable cells (MCF-7-Control and MCF-7-antimiR-203) were suspended in 100 μl serum free medium and injected into mammary fat pad (*n* = 5). Tumor volume was measured using digital caliper till the end of experiments. Tumor volume was calculated according to the formula L × W^2^ × 0.5 (L = length; W = width; all parameters in millimeters). After sacrificing, a portion of the tumor was snap-frozen and stored at −80°C for biochemical analysis.

### Statistical analysis

Results were expressed as the mean ± standard deviation (SD), and statistical analyses were performed using two-tailed paired or unpaired Student*t*test in GraphPad Prism 6 (GraphPad, La Jolla, CA). A*p*value of < 0.05 was considered statistically significant.
